# Fatigue-Related and Timescale-Dependent Changes in Individual Movement Patterns Identified Using Support Vector Machine

**DOI:** 10.3389/fpsyg.2020.551548

**Published:** 2020-09-30

**Authors:** Johannes Burdack, Fabian Horst, Daniel Aragonés, Alexander Eekhoff, Wolfgang Immanuel Schöllhorn

**Affiliations:** ^1^Department of Training and Movement Science, Institute of Sports Science, Johannes Gutenberg University Mainz, Mainz, Germany; ^2^Department of Wushu, School of Martial Arts, Shanghai University of Sport, Shanghai, China

**Keywords:** situatedness, individuality, kinematic data, optimal movement, fatigue, support vector machine, machine learning, movement classification

## Abstract

The scientific and practical fields—especially high-performance sports—increasingly request a stronger focus be placed on individual athletes in human movement science research. Machine learning methods have shown efficacy in this context by identifying the unique movement patterns of individuals and distinguishing their intra-individual changes over time. The objective of this investigation is to analyze biomechanically described movement patterns during the fatigue-related accumulation process within a single training session of a high number of repeated executions of a ballistic sports movement—specifically, the frontal foot kick (*mae-geri*) in karate—in expert athletes. The two leading research questions presented for consideration are (1) Can characteristics of individual movement patterns be observed throughout the entire training session despite continuous changes, i.e., even as fatigue-related processes increase? and (2) How do intra-individual movement patterns change as fatigue-related processes increase throughout a training session? Sixteen expert karatekas performed 606 frontal foot kicks directed toward an imaginary target. The kicks were performed in nine sets at 80% (*K*-80) of the self-experienced maximal intensity. In addition, six kicks at maximal intensity (*K*-100) were performed after each of the nine sets. Between the sets, the participants took a 90-s break. Three-dimensional full-body kinematic data of all kicks were recorded with 10 infrared cameras. The normalized waveforms of nine upper- and lower-body joint angles were classified using a supervised machine learning method (support vector machine). The results of the classification revealed a disjunct distinction between the kinematic movement patterns of individual athletes. The identification of unique movement patterns of individual athletes was independent of the intensity and the degree of fatigue-related processes. In other words, even with the accumulation of fatigue-related processes, the unique movement patterns of an individual athlete can be clearly identified. During the training session, changes in intra-individual movement patterns could also be detected, indicating the occurrence of adaptations in individual movement patterns throughout the fatigue-related accumulation process. The results suggest that these adaptations can be modeled in terms of changes in patterns rather than increasing variance. Practical consequences are critically discussed.

## Introduction

Since the beginnings of sports science in the eastern and western hemispheres, quantitative analyses of the athletes’ momentary performance have been performed, targeted toward attaining future improvements and optimization (Matwejew, 1972; Hay, 1978). Following the quantification trend in biomechanics and learning psychology since the 1960s, versatile attempts were made to continuously approach previously established target values by applying control loop models (Anochin, 1935; Miller et al., 1960). Typically for this purpose, group averages of the world’s best athletes were chosen to serve as target values and, thus, as a collective orientation for sports training. Medically (Hollmann and Hettinger, 2000; Kjaer et al., 2003) and biomechanically (Hay, 1978) based conditioning and coordination profiles were drawn up, which had to be copied by athletes with extensive numbers of executions of the movement tasks and correction processes (Harre, 1969; Matwejew, 1972; Letzelter, 1978; Martin et al., 1991). Driven by the idea to improve the monitoring and control of sports training, increasingly precise measurement methods for describing human movements were developed. Consequently, fluctuations in movement data also became more obvious. Although anecdotal evidence (Bernstein, 1967) and theoretical considerations (Hatze, 1986) presented early on pointed to the non-repeatability of movements, fluctuations between and within individuals were, for a long time, mostly regarded as measurement errors or as destructive noise. Changes in sports training philosophies were rarely observed.

Over time, however, increasing doubts (Nubar and Contini, 1961; Beckett and Chang, 1968; Hatze, 1973, 1984, 1986) about the orientation of collective (person-independent) profiles eventually led to the development of more group-specific profiles as a basis for orientation for sports training—for example, profiles established according to age, gender, or anthropometry. Later, the availability of more powerful computers enabled the biomechanical simulation of coordination profiles optimized for individual athletes, including those based on person-specific anthropometric characteristics and/or isometric force values (Winter, 1980; Gutewort and Sust, 1989; Liu, 1992; van Soest et al., 1993; Nigg, 1994). The effort to orientate sports training more toward assisting individual athletes rather than toward benefiting the collective (person-independent) average profiles was supported by findings that allowed researchers to distinguish world-class athletes based on their metabolic adaptation behavior (Bouchard and Rankinen, 2001), their muscle-related strength abilities (Sust and Jung, 1990; Weiss et al., 1995), and their movement patterns (Bauer and Schöllhorn, 1997; Schöllhorn and Bauer, 1997, 1998). However, two challenges that were considered to help maintain a persistent gap between theory and practice were not resolved: one concerned the question of whether and how athletes are able to perform according to profiles that were theoretically predicted as being optimal for them, while the other related to the enormous adaptability of the human movement system and the permanent fluctuations of human movement behavior.

An integrated approach was suggested to address these challenges by linking two previously largely separated fields of research, sports biomechanics (Nigg, 1994; Winter, 2009) and system dynamics (Schöner et al., 1986). This involved, on the one hand, parallel observation of fluctuations of various biomechanical variables that describe the behavior of individual athletes in longitudinal studies (Schöllhorn, 1993; Schöllhorn and Bauer, 1997; Schöllhorn et al., 2001), and, on the other hand, fluctuations as an essential feature of dissipative systems in adaptation processes (Schöner et al., 1986). Another contributing issue was related to a principle of biomechanically supported training control (Farfel, 1977; Ballreich et al., 1986), according to which the effect of a variable identified as influencing the overall performance is estimated by its systematic variation. Two major consequences were connected to this linking of sports biomechanics and system dynamics. On the one hand, fluctuations in biomechanically controlled training became reinterpreted and were used for initiating a self-organizing process through their amplification (Schöllhorn, 2000). Variable exercises and deviations caused by internal (e.g., fatigue, emotion, and kinematics), external (e.g., ball weight, field size, and number of team mates), and entangled (e.g., gravitational forces and perception) influencing factors were no more considered as independent or destructive but rather as tools for modifying the athlete’s or learner’s potential. On the other hand, increased efforts were observed toward realizing the application of pattern recognition methods for a more detailed analysis of the interdependence of individual movement patterns and its fluctuations. Based on methods gleaned from the research areas of artificial intelligence and machine learning, “patterns” should be identified in the fluctuations of time-continuous waveforms of biomechanical variables.

First applications of machine learning methods in the field of sports and everyday movements resulted in the identification of individuals based on their disjunct movement patterns during gait (Nixon et al., 1999; Schöllhorn et al., 1999, 2002), running (Simon and Schöllhorn, 1995), pole-vaulting (Jaitner and Schöllhorn, 1995), discus-throwing (Bauer and Schöllhorn, 1997), and javelin-throwing (Schöllhorn and Bauer, 1998). Besides the identification of individual movement patterns even within world-class athletes, who have already experienced thousands of executions in their sports discipline and formerly served as collective profiles for sports training, permanent fluctuations in the biomechanical movement patterns, no matter whether time-discrete or time-continuous, supported earlier evidence of an extremely low probability of identical movement patterns existing between multiple executions of a movement task (Bauer and Schöllhorn, 1997; Schöllhorn and Bauer, 1997, 1998).

Following the differentiation of individual movement patterns, emotion-specific (Janssen et al., 2008) or fatigue-specific (Jäger et al., 2003; Janssen et al., 2011) subpatterns could be identified within individual movement patterns. However, classifications were made based on pre- and post measurements, while the actual process of becoming fatigued or the actual process of changes trending toward a specific emotional state was not investigated. A further step toward an even more differentiated analysis of fluctuations in biomechanical variables can be assigned to recent findings of highly time-dependent movement patterns. Disjunct changes in individual movement patterns without any intervention (Horst et al., 2016, 2017a) indicate permanent adaptations of the movement system. For example, kinematic gait patterns of the same person could be distinguished within 1 day after a 30-min break with a classification accuracy of 91% (Horst et al., 2017a), while the classification accuracy between days was 98% (Horst et al., 2016). Despite permanent disjunct changes in individual movement patterns over time (Horst et al., 2016, 2017a) and the “non-repeatability” of movement patterns overall (Bernstein, 1967; Hatze, 1986; Newell and Corcos, 1993), unique movement patterns of individual people could be identified even 1 year later (Horst et al., 2017b).

Overall, the pattern-recognition approach introduced for differentiated movement analysis using machine learning methods provides promising insights not only regarding individuals and whole-body movements on a rather coarse scale of observation but, also, the analysis of fluctuations within individuals on a finer scale. Neither emotion-specific nor daily changes of movement patterns have found equivalents in biomechanical simulation modeling so far (Glazier and Mehdizadeh, 2019a,b).

To what extent and at what kind of timescale do the fluctuations of movement patterns change or shift by means of fatiguing training in such a way that the identification of individuality is disturbed by a disjunct separation of the variance-related distributions is the subject of this work. Considering a typical karate training session (Funakoshi, 1973), we conducted a biomechanical movement analysis of a large number of executions of a frontal kick task during a fatiguing process. Fatigue is a naturally occurring influence of movement adjustments inherent in any training session or competition. While the influence of fatigue on performance measures has been well-described (Enoka and Stuart, 1992; Gandevia, 2001), the detailed effects of fatigue on movement execution are only partially elucidated. Most studies to date on the influence of fatigue on movements have been conducted focusing on basic cyclic movements (e.g., walking and cycling), while only a few have focused on ballistic movements and considering just a small number of actions. In these studies, the occurrence of spontaneous movement adjustments under fatigue as a result of multiple executions in various disciplines was reported, including rope-skipping (Bruce et al., 2017), running (Mizrahi et al., 2000), water polo (Oliveira et al., 2016), football (Amiri-Khorasani et al., 2011), and karate (Quinzi et al., 2016).

Aragonés et al. (2018) investigated fatigue-related changes of kinematics at different timescales during a karate training session consisting of many frontal foot kicks. The resulting data contained evidence of timescale-dependent adjustments in kicking patterns occurring, particularly during the first 20 executions on a timescale of some tens of seconds (Quinzi et al., 2016; Aragonés et al., 2018). On the same timescale, mainly variables related to the speed of the movement and their relative maxima changed, while variables related to the form of the kicking movement were hardly affected. However, when using the timescale of tens of minutes, exactly the opposite was noticeable. Understanding fatigue-related movement changes according to different timescales is of great relevance in applied biomechanics since exercise-related fatigue is a source of temporary change that introduces its own timescale into performance dynamics (Newell et al., 2009). In sum, a clear and current deficit in the understanding of timescale-dependent changes in movements can be stated. Previous studies have mostly considered discrete biomechanical variables at discrete time points. To our knowledge, an analysis of sports movement patterns based on time-continuous biomechanical variables over many executions of the same movement task has not been conducted so far.

In this study, the karate front kick is used exemplarily to examine a ballistic whole-body movement by means of pattern recognition procedures (i.e., support vector machine) on the one hand with regard to its individuality and on the other hand with regard to its situatedness over a fatiguing process. Situatedness here refers to spatiotemporal contingency as the momentary being that not only results from environmental, but also from—for example—sociocultural, geographical, historical, and biographical conditions as has been introduced in phenomenology (Heidegger, 1927; Merleau-Ponty, 1945). Thereby, the leading research questions of this investigation are (1) can characteristics of individual movement patterns be observed throughout the entire training session despite continuous changes, i.e., even as fatigue-related processes increase? and (2) how do intra-individual movement patterns change as fatigue-related processes increase throughout a single training session?

## Materials and Methods

The present analysis was conducted on data collected by Aragonés et al. (2018). The application of machine learning methods for classification offers an extended perspective on the data and provides a more differentiated insight into the development of the foot-kick kinematics of individual athletes over two distinguishable timescales.

### Participants and Ethics Statement

The study participants were 16 Caucasian healthy adults (11 men and five women) who practiced karate at least twice a week (the group characteristics are shown in Table 1). All were right-footed and were expert karatekas with brown and black belts from the first to the fifth dan. The participants were recruited from local karate clubs, and all were practicing karate at the time of the examination for recreational and health purposes. Before participating in the study, the participants signed informed consent forms. All experimental procedures were conducted in accordance with the Declaration of Helsinki and were approved by the ethical committee of the medical association Rhineland-Palatinate in Mainz. Each participant visited the biomechanics laboratory once, where all kinematic measurements took place.

**TABLE 1:** Participant characteristics.

	M	SD
Age (years)	39.69	12.81
Height (m)	1.75	0.08
Body mass (kg)	73.14	12.33
BMI (kg/m^2^)	24.54	3.11
Experience in karate (years)	16.00	6.60
Left leg length (m)	0.81	0.05
Right leg length (m)	0.81	0.05

*Data are presented as mean (M) and standard deviation (SD), BMI, body mass index.*

### Experimental Protocol

**FIGURE 1:**
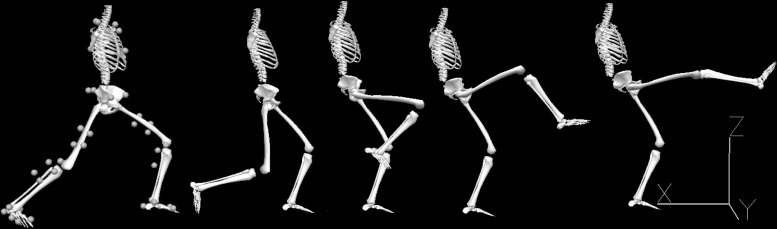
Biomechanical model representing the *zenkutsu-dachi* starting stance (with 42 retroreflective markers fixed on anatomical landmarks) and the *mae-geri* kick sequence performed with the right leg. Adapted from Aragonés et al. (2018).

The karate front kick (i.e., *mae-geri-keage*; see Figure 1) was the movement to be performed before returning immediately to the starting position. The kick was directed without impact at a reference target (0.1 m × 0.1 m) supported by a plastic rod placed 3 m in front of the participant and adjusted to the participant’s abdominal height. Using a metronome, the actions were prompted acoustically at a frequency corresponding to a kick every 2 s (the participants changed from the orthodox to the southpaw stance and vice versa for 2 s). The starting stance was *zenkutsu-dachi* (Figure 1), that is, standing with one foot in front of the other without lifting the heels from the mat. One front and one rear martial arts mat (size: 0.9 m × 0.6 m; height: 0.02 m; material: foam rubber; surface: rice straw pattern) had previously been attached to the floor in such a way that each foot was placed on one of the mats. The participants were asked to keep the angle between the thigh and lower leg segments of the front leg at around 135°, and to keep the rear leg extended as far as possible. The lateral distance between both feet corresponded to the width of the pelvis.

**FIGURE 2:**
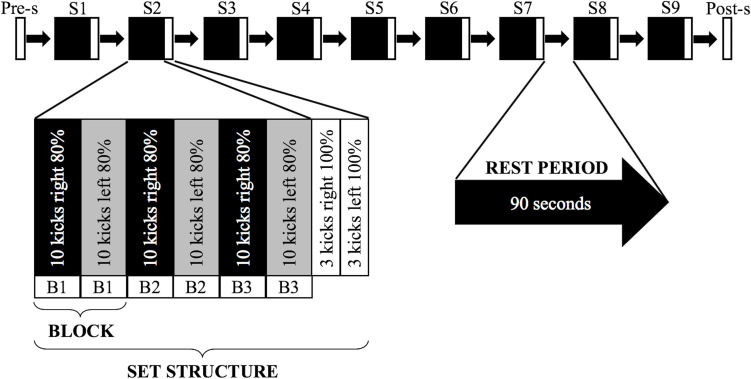
Schematic of the test protocol. The experimental procedure consisted of one pre-set (Pre-s) followed by nine sets (S1–S9) with 90-s rest intervals in between and an additional post-set (Post-s) performed after a 10-min rest period. A deeper insight into the structure of the main elements is represented below. The pre-set and post-set consisted of six *mae-geri* kicks performed at maximal intensity (*K*-100). Adapted from Aragonés et al. (2018).

The test protocol was developed in the style of a common karate training protocol (Funakoshi, 1973), where participants are often asked to perform dozens of executions at submaximal intensity before finally performing a few executions at maximal intensity. Such bouts alternate with short intervals of inactivity, during which time, the teacher gives corrections. As shown in a schematic presentation of the test protocol in Figure 2, the participants were asked to perform nine sets of kicks, each consisting of 60 kicks with 80% of their self-perceived maximal intensity (*K*-80) (three blocks of 10 kicks alternately with each leg, starting with the right leg), followed by one set of six kicks at maximal intensity (*K*-100) (three with the right leg, then three with the left leg). Before set 1 and after set 9, participants were asked to perform a pre-set and a post-set of six kicks at maximal intensity (*K*-100) (three with the right leg, then three with the left leg). Between the sets, the participants rested for 90 s, except between set 9 and the post-set, when they rested for 10 min. The break of 90 s corresponds on the one hand to the typical break length of karate training, and on the other hand, it was needed to collect the physiological and psychological variables as well as to readjust any markers that might have become loose during prior sets.

After the participants arrived at the laboratory and before the test was initiated, passive optical markers for the purpose of biomechanical analysis were attached at anatomical landmarks. In addition, discrete measurements of heart rate (HR), blood lactate concentration, and the rating of perceived exertion (RPE) were performed to collect baseline values. The participants were then introduced to the test protocol and encouraged to warm up by performing (1) 5 min of self-directed warm-up and (2) one set of kicks while reporting the RPE (they were already familiar with the appropriate scale) to become accustomed to the protocol.

### Data Acquisition

#### Physiological: Heart Rate and Blood Lactate Concentration

A heart rate (HR) monitor attached to a chest strap (HRM2-SS; Garmin, Schaffhausen, Switzerland) and a blood lactate concentration analyzer (h/p/cosmos sirius; SensLab, Leipzig, Germany) were used.

#### Psychological: Borg’s Rating of Perceived Exertion

Borg’s rating of perceived exertion (RPE) on a scale of six to 20 points and corresponding instructions (Borg, 1998) was adopted.

#### Kinematics

Kinematic data were recorded with 10 infrared cameras (Oqus 310; Qualisys, Gothenburg, Sweden), which recorded at a frequency of 333 Hz. Forty-two retroreflective markers were attached to anatomical landmarks (Figure 1), including the left and right anterior superior iliac spine, the left and right posterior superior iliac spine, the right femur laterally and medially, the left and right fibula tip of the lateral malleolus, the left and right tibia tip of the medial malleolus, the left and right head of the first metatarsus, the left and right head of the fifth metatarsus, the tuberosity of the fifth metatarsus, the posterior surface of the calcaneus, the left and right acromion, the sternum jugular notch, the sternum xiphisternal joint, the seventh cervical vertebrae, and the midpoint between the inferior angles of most caudal points of the two scapulas. Two clusters of four markers were fixed to the lateral sides of the left and right thighs and the left and right shanks, respectively.

### Data Processing

#### Physiological and Psychological Variables

Discrete HR values were recorded, blood lactate concentration samples were taken from the earlobe, and RPE values were reported by the participants before the warm-up phase (i.e., baseline values), after the pre-set, after each of the nine sets during the rest period, and after the post-set, respectively. These variables were selected to monitor fatigue development.

#### Kinematic Variables

The kicks were analyzed from the moment the kicking foot moved forward on the x-axis to the maximum knee extension angle just before the leg returned (Figure 1). The marker trajectories were low-pass filtered with a sixth-order Butterworth zero-phase filter with a cut-off frequency of 15 Hz. A partial body model, based on the standard segments of the foot, shank, thigh, thorax, and the CODA pelvis segment (Charnwood Dynamics, Rothley, United Kingdom), was created for each participant in the standing position using Visual 3D Standard version 4.86.0 (C-Motion, Germantown, MD, United States). The joint angles were calculated with a Cardan sequence of rotation (Cole et al., 1993). The data were processed with Matlab version R2015b (The Mathworks, Natick, MA, United States). All variables were time-normalized to 101 data points, z-normalized, and scaled to the range (−1, 1). The following nine joint angle waveforms were calculated in the *x*-, *y*-, and *z*-planes: the left and right ankle joint angle, the left and right knee joint angle, the left and right hip joint angle, the sternoclavicular joint angle, and the angles between the left and the right thighs to the thorax.

### Data Analysis

#### Data Classification

The classification of karate patterns was based on 606 kicks [606 = 2 (left kicks + right kicks) * 303 (270 *K*-80 + 33 *K*-100)] performed by each participant. For each kick, a concatenated vector of all 27 kinematic variables [2727 = 27 joint angle waveforms (9 joint and segment angles in the *x*-, *y*-, and *z*-planes) ^∗^ 101 data points] was built and used for classification purposes. The classification was based on a support vector machine, supervised machine-learning classifier (Boser et al., 1992; Cortes and Vapnik, 1995; Müller et al., 2001; Schölkopf and Smola, 2002) using a linear kernel and a grid search to determine the best cost parameter (*C* = 2^–5^, 2^–4.75^,…, 2^15^). The ability to distinguish karate patterns between participants (16-class classification) and within a participant between different combinations of blocks and sets (27-class classification), sets (nine-class classification), and blocks (three-class classification) was investigated in a multiclass classification setting. As presented in Table 2, due to the different classification tasks, the size of the matrices used for the classifications differed. Therefore, the prediction accuracies, F_1_ scores, precision, and recall were calculated over a *k*-fold cross-validation depending on the minimal number of kicks included in a block in each classification task. Furthermore, for every classification task, the data were divided into training and test groups. The data in the test group were evenly distributed across all classes. This splitting of the data was stratified repeatedly depending on the number of sets [i.e., by participant (*K*-80 and *K*-100) and block-within-set classifications] and the minimal number of kicks in one block (i.e., block and set classifications) to obtain meaningful results. This procedure ensured that each kick was included in every classification task exactly once in the test set, thereby avoiding random imbalances in the prediction and making the results more reproducible. The classification was performed within Python version 3.6.3 (Python Software Foundation, Wilmington, DE, United States) using the scikit-learn toolbox (version 0.22.1) (Pedregosa et al., 2011).

**TABLE 2:** Description of the input data and validation procedure depending on the different classification tasks.

Classification task and intensity	Size of matrix	Description of *x*-vector length	Training and test groups	Cross-validation * stratified splitting	Number of classes
Participant *K*-80	4320 × 2727	4320 = 16 participants * 270 kicks	Training: 16 participants * 30 kicks * 8 sets (= 3840 kicks) Test: 16 participants * 30 kicks * 1 set (= 480 kicks)	9-fold * 9	16

Participant *K*-100	528 × 2727	528 = 16 participants * 33 kicks	Training: 16 participants * 3 kicks * 10 sets (= 480 kicks)	3-fold * 11	16
			Test: 16 participants * 3 kicks * 1 set (= 48 kicks)		

Block *K*-80	270 × 2727	270 = 27 combinations of sets and blocks (9 sets * 3 blocks) * 10 kicks	Training: 9 kicks * 27 blocks (= 243 kicks) Test: 1 kick * 27 blocks (= 27 kicks)	9-fold * 9	27

Set *K*-80	270 × 2727	270 = 9 sets * 30 kicks	Training: 27 kicks (9 per block) * 9 sets (= 243 kicks)	9-fold * 9	9
			Test: 3 kicks (1 per block) * 9 sets (27 kicks)		

Block-within-set *K*-80	270 × 2727	270 = 3 Blocks * 9 sets * 10 kicks	Training: 10 kicks * 3 blocks * 8 sets (= 270 kicks)	9-fold * 9	3
			Test: 10 kicks * 3 blocks * 1 set		

Kicks for classification are based on the left- or the right-footed kicks; 2727 = 1 leg * 27 joint angle waveforms (x-, y-, and z-plane * 9 joint angles) * 101 data points.

#### Statistical Analysis of Movement Variance, Physiological, and Psychological Variables

To determine the variance of movement patterns over time, the coefficient of variation (CV) was calculated over the joint angle waveforms of each participant (Winter, 1984). The exact same data were used to calculate the CV as those used for the classification analysis described above. The CV was calculated according to each classification task. This means that, according to the inter-individual classification, the CV was calculated over the waveforms of all participants; in other words, according to intra-individual classification tasks in the block and set classifications over one block or one set of a participant as well as in the block-within-set classification over all three blocks within the sets.

The CV according to each classification problem, the HR, the lactate blood concentration, and the RPE were tested for normal distribution using the Shapiro–Wilk test. For data that did not deviate significantly from the normal distribution, descriptive statistics are presented in means and standard deviations (SDs). Statistical analysis was performed using repeated-measures analysis of variance (RM-ANOVA) with *post hoc* paired *t*-tests with Holm–Bonferroni correction. Data that deviated significantly from the normal distribution were statistically tested with Friedman ANOVA; *post hoc* analysis was performed with the Wilcoxon paired-rank test with Holm–Bonferroni correction. The results were considered significant at *p* < 0.05. Effect size was tested with η^2^ eta-squared for the RM-ANOVA, Cohen’s *d* for the *t*-test, and r-effect size for the Wilcoxon test, respectively. The analyses were performed using the Statistical Package for the Social Sciences version 23 software program (IBM Corporation, Armonk, NY, United States).

## Results

### Inter-Individual Classification

As presented in Table 3, the classification of movement patterns between the participants achieved 100% accuracy at both the maximal (*K*-100) and submaximal (*K*-80) intensities during the karate training process. The movement patterns of the participants can, therefore, be clearly distinguished. Only one left kick (1/4,320) and one right kick (1/4,320) at submaximal intensity were not correctly classified.

**TABLE 3:** Mean percentage values of accuracies, *F*_1_ scores, precision scores, and recall scores of the different classification tasks and corresponding CVs.

		Accuracy (%)		*F*_1_ Score (%)		Precision (%)		Recall (%)		Number of classes	Random baseline accuracy (%)	CV (%)	
								
Classification task and kick intensity	Leg	*M*	*SD*	*M*	*SD*	*M*	*SD*	*M*	*SD*			*M*	*SD*
Participant *K*-80	Left	100.0	0.1	100.0	0.1	100.0	0.1	100.0	0.1	16	6.3	31.6	
	Right	100.0	0.1	100.0	0.1	100.0	0.1	100.0	0.1	16	6.3	32.2	

Participant *K*-100	Left	100.0	0.0	100.0	0.0	100.0	0.0	100.0	0.0	16	6.3	32.1	
	Right	100.0	0.0	100.0	0.0	100.0	0.0	100.0	0.0	16	6.3	33.3	

Block *K*-80	Left	42.9	2.3	52.3	2.4	48.1	2.5	62.0	2.5	27	3.7	16.3	2.4
	Right	42.9	1.8	52.2	1.9	47.7	1.6	62.5	2.7	27	3.7	16.6	2.2

Set *K*-80	Left	66.2	2.7	66.0	2.9	69.2	3.5	67.4	2.6	9	11.1	16.7	2.2
	Right	65.1	2.6	64.7	2.8	68.7	3.1	66.2	2.7	9	11.1	17.2	1.9

Block-within-set *K*-80	Left	55.6	3.6	54.5	4.0	57.3	3.9	56.3	3.0	3	33.3	17.9	1.9
	Right	66.0	1.3	64.9	1.8	68.1	1.1	66.3	1.4	3	33.3	18.3	1.7

*Mean (M) and standard deviation (SD) values of the classification rates calculated over the different training–test splits (Table 2). M and SD values of the CV over the waveforms for each classification task; for block, set, and block-within-set classification, M and SD values for all participants and classes are presented.*

### Intra-Individual Classification

**FIGURE 3:**
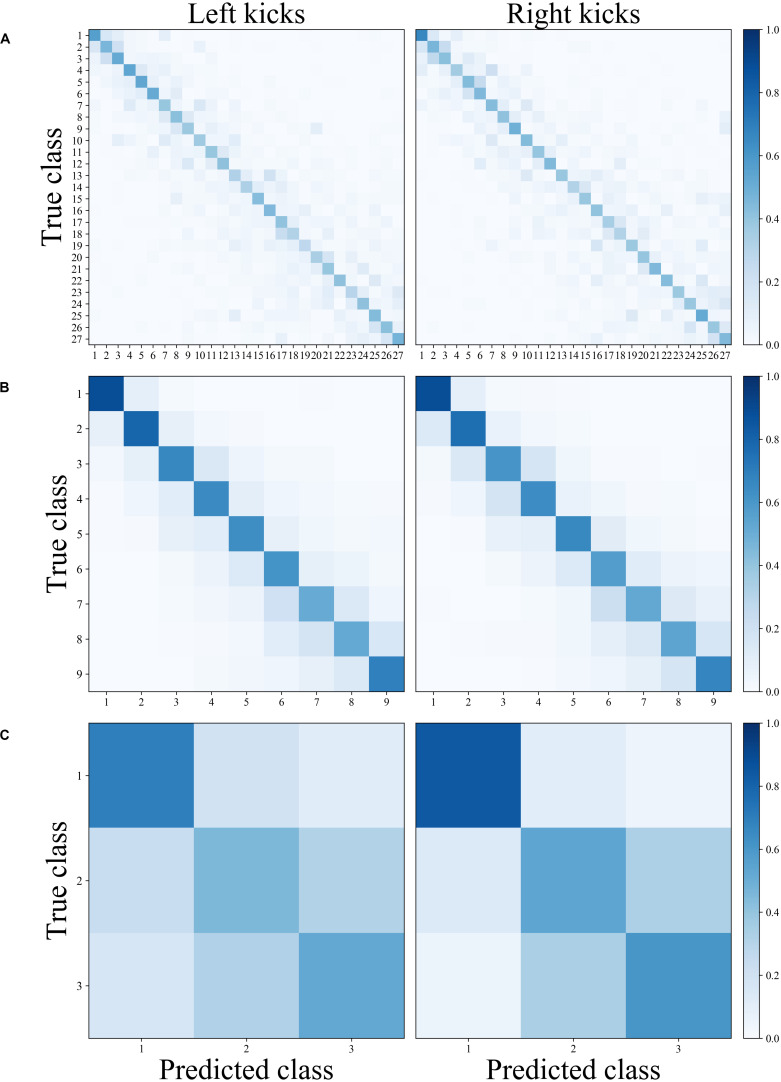
Normalized confusion matrices of the classification’s movement patterns of the left and right kicks depending on the different classification tasks. **(A)** Block classification, **(B)** set classification, and **(C)** block-within-set classification.

As shown in Table 3, the classification of movement patterns over the 27 blocks in all sets (27 classes) resulted in a prediction accuracy of 42.9% ± 2.3% for the left kicks and 42.9% ± 1.8% for the right kicks; the classification of the movement patterns of the nine sets (nine classes) resulted in a prediction accuracy of 66.2% ± 2.7% for the left kicks and 65.1% ± 2.6 for the right kicks, and the classification of the movement patterns of the three blocks within the sets (three classes) resulted in a prediction accuracy of 55.6% ± 3.6% for the left kicks and 66.0% ± 1.3% for the right kicks. Figure 3 shows the confusion matrices of the right and left kicks for all intra-individual classifications. It is noticeable that the true class has always been predicted more often and that the misclassifications are mainly distributed among the classes nearby.

**FIGURE 4:**
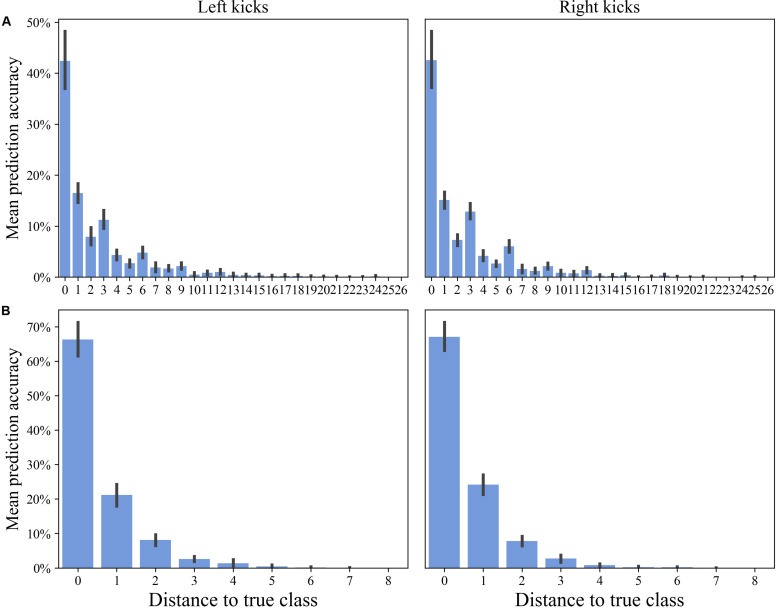
Distances to true classes of the classification of the movement patterns of the left and right kicks depending on the classification task. Presented here are the means and SDs for each distance. A distance of zero to the true class refers to the prediction accuracy of the true class (e.g., a distance of two means that the predicted class is two classes next to the true class). **(A)** Block classification. Consider that distances of 14 and higher will not occur in all cases (e.g., block 14 has only 13 blocks before and 13 after). **(B)** Set classification. Consider that distances of five and higher will not occur in all cases.

This is also displayed in Figure 4, where the mean prediction accuracy is shown as a function of the distance to the true class. For both the left and right kicks, the true class was the most probable class, and the probability of prediction tends to decrease with increasing distance from the class. It is noticeable, however, that groups with a distance of three and multiples of three blocks again exhibit a higher probability than that of the class closer to them. A distance of three classes means that the class corresponds to the same block in the nearby set. Six classes correspond accordingly to the same block only with the distance of two sets between them. However, the set classification clearly shows that the probability of a misclassification decreases significantly with increasing distance of a class from the true class.

### Inter- and Intra-Individual CV

**FIGURE 5:**
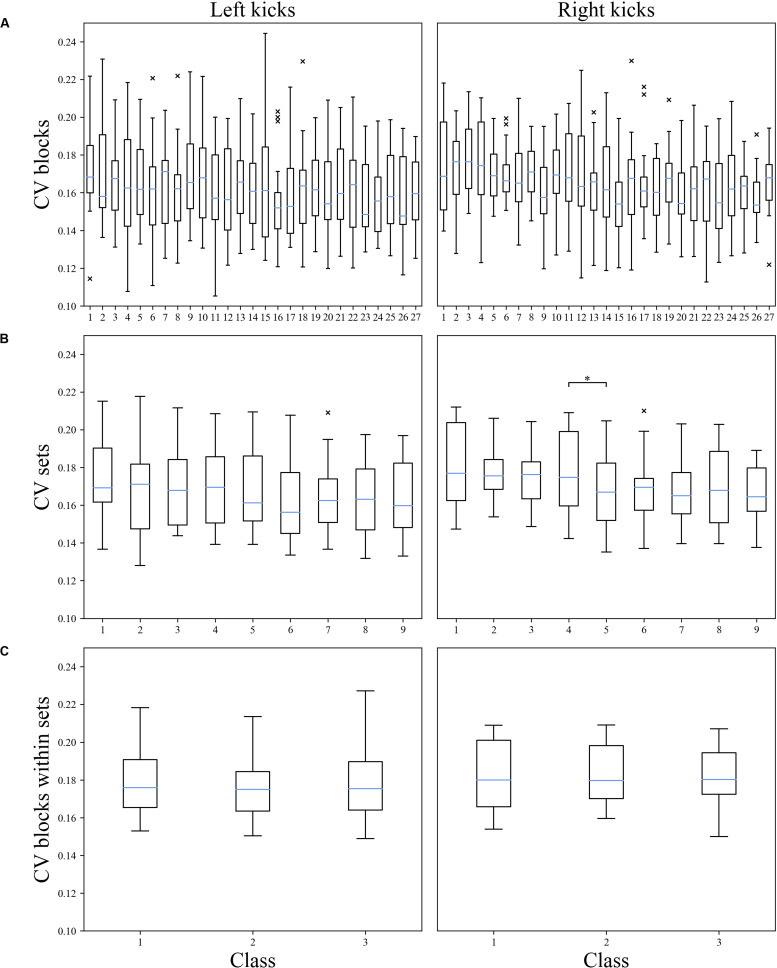
Coefficient of variations of the joint angle waveform of the left and right kicks depending on each block and set. Presented here are the box plots for all participants; the *x*-axis shows the classes, and the *y*-axis shows the CV. Values are considered as outliers if they are outside the interval [*Q*_1_ – 1.5 * (*Q*_3_ – *Q*_1_), *Q*_3_ + 1.5 * (*Q*_3_ – *Q*_1_)]. × = Outlier (each × stands for one outlier); *Statistically significant difference in pairwise *post hoc* test. **(A)** CV of the waveforms of the blocks of each participant. There was a statistically significant result for right kicks. **(B)** CV of the waveforms of the sets of each participant. There was a statistically significant result for right kicks. **(C)** CV of the waveforms of the blocks within sets for all participants.

As presented in Table 3, the inter-individual CVs of the waveforms of kicks with 80% intensity were 31.6% for the left kicks and 32.2% for the right kicks. The CVs of the waveforms of the kicks performed with maximal intensity using the left leg (32.1%) and the right leg (33.3%) are slightly higher. The mean CVs of the waveforms of the intra-individual comparisons, therefore, are lower, with values between 16.3 and 18.3%. What is noticeable here is that the CV of the waveforms of the right kicks is always slightly higher than that of the left kicks. Figure 5 shows the CVs of waveforms dependent on blocks, sets, or blocks within sets. It was noticeable that the CV values of waveforms did not increase in any case during the fatigue-accumulating process. As shown in Table 4, the statistical comparisons of the CVs within the blocks and within the sets reveal a significant difference for the waveforms of the right kicks, while the CVs of the waveforms of the left kicks do not differ significantly. In paired *post hoc* comparisons only between sets 4 and 5, there was a statistically significant difference noted among the right kicks. Based on the descriptive CV values for the waveforms of the blocks and sets, it may be stated that the values for the first block and set do not increase.

**TABLE 4:** Statistical analysis of the intra-personal CV of the joint angle waveforms of the *K*-80 kicks.

Classification task	Leg	RM-ANOVA or Friedman ANOVA	*Post hoc* analysis
Block	Left	χ*^2^(*26) = 37.881, *p* = 0.062	
	Right	χ^2^(26) = 54.696, *p* = 0.001^a^	

Set	Left	*F*(3.933,59) = 2.212, *p* = 0.080, η^2^ = 0.129	Sets 4–5
	Right	*F*(8,120) = 4.462, *p* < 0.001^a^, η^2^ = 0.229	[*p*(15) = 0.0003^b^, *d* = 0.499]

Block-within-set	Left	*F*(2,30) = 0.607, *p* = 0.552, η^2^ = 0.039	
	Right	*F*(2,30) = 0.119, *p* = 0.888, η^2^ = 0.008	

*Presented here are the results of the RM-ANOVA or the Friedman ANOVA to determine differences among all classes. Only the significant post hoc paired-samples t-test or Wilcoxon signed-rank test results are shown. ^*a*^Significance level: α = 0.05. ^*b*^Holm–Bonferroni-corrected significance level: α_0_ = 0.0016.*

### Changes in Physical and Psychological Variables Between Sets

**FIGURE 6:**
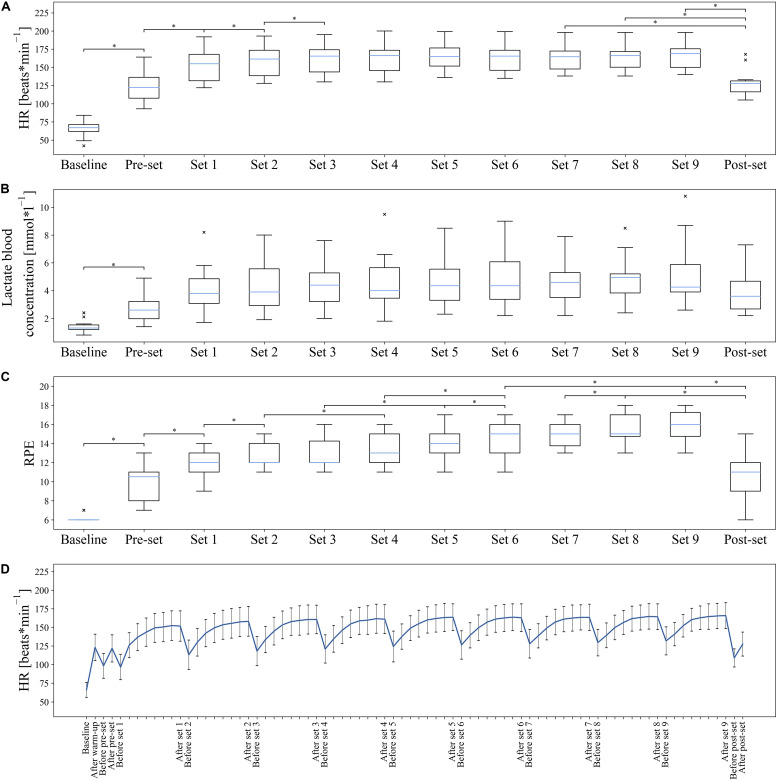
Development of HR, lactate blood concentration, and RPE over the duration of the examination. Values are considered as outliers if they are outside the interval [*Q*_1_ – 1.5 * (*Q*_3_ – *Q*_1_), *Q*_3_ + 1.5 * (*Q*_3_ – *Q*_1_)]. × = outlier (each × stands for one outlier); *Statistically significant difference in pairwise *post hoc* test. **(A–C)** Shown are the box plots of HR, lactate blood concentration, and RPE from baseline measurement and according to the respective sets. HR, lactate blood concentration, and RPE showed significant results. For reasons of clarity, only significant adjacent pairs of *post hoc* comparisons are shown. If the difference to the nearest neighbor was not significant, the difference to the next but one neighbor was shown. If this difference was not significant either, the next neighbor was shown. An overview of all *post hoc* comparisons is presented in Supplementary Table S1. **(D)** The mean values and SDs of the continuous HR curve are shown.

The baseline values [median (interquartile range)] for HR, lactate blood concentration and RPE were 67 (61.75–71.25) beats * min^–1^, 1.30 (1.20–1.53) mmol * l^–1^, and 6 (6–6). Both HR [χ^2^(11) = 133.520; *p* < 0.001], lactate blood concentration [χ^2^(11) = 77.768; *p* < 0.001], and RPE [χ^2^(11) = 161.988; *p* < 0.001] showed statistically significant differences over the course of the experiment with fatigue accumulation from baseline through the time points immediately following each set (Figure 6). All results of Friedman ANOVAs and Wilcoxon signed-rank *post hoc* tests are presented in Supplementary Table S1. It was noticeable that the HR increased steadily over the course of a set and decreased by about 30–40 beats * min^–1^ in the 90-s set pauses. The maximum HR reached a median of 169 (150.25–175.50) beats * min^–1^ after the ninth set. A statistical comparison of the times directly after the completion of each set and the baseline measurement showed a significant increase until after the third set. There were no statistical differences between the third and ninth set, with the median HR over the course of the test increasing from 165 to 169 beats * min^–1^. The analysis of the blood lactate concentration showed only statistical differences between pairs of baseline measurements and all further sets. No statistical differences were found between the individual sets. However, up to the end of set 8, a trend can be observed that the mean lactate value increased continuously and reached a maximum of 4.95 (3.83–5.20) mmol * l^–1^. In pairwise comparisons of the RPE, a steady increase was observed until the end of the ninth set. It is shown that the RPE increases significantly at the next, the next but one, or at the latest the third following set and reaches a maximum of 16 (14.75–17.25).

## Discussion

In this study, the biomechanical movement patterns of experts in karate were investigated by executing the front kick, constituting a movement performed with a high level of expertise, multiple times, and through a fatigue-accumulating process in a training session. In relation to the first hypothesis, the results show that an individual’s movement patterns can be clearly identified independently of fatigue-accumulating training. Regarding the second hypothesis, it was found that changes in the intra-individual movement patterns, which are not attributable to changes in variance, can be clearly identified within a training session. In detail, these changes in movement patterns appear to be dependent on different timescales. Whether these timescales are independent of each other, contain self-similar features, or correspond to timescales related to adaptation, warming up, and learning (Newell et al., 2001) will challenge future research.

### Individuality of Movement Patterns

The results of the classification analysis showed that the movement patterns of all 16 participants could be clearly distinguished from each other, although all tried to imitate the same profile of the frontal foot kick (*mae-geri*). Unique movement patterns could be distinguished for each participant for both kicks performed at both maximal (*K*-100) and submaximal (*K*-80) intensities as well as with regard to the respective leg performing the kick. Interestingly, the unique characteristics of the individual movement patterns could be identified throughout the entire training session, despite breaks and the accumulation of fatigue. The results support the idea that detailed adaptations of movement patterns to new situations should only be sought on an individual level and not on the basis of a collective, person-independent profiles (Schöllhorn, 1993; Schöllhorn and Bauer, 1998; Janssen et al., 2011; Eekhoff et al., 2016; Horst et al., 2017b; Glazier and Mehdizadeh, 2019a,b). Despite all the fluctuations apparent in the kinematic variable waveforms, which were superimposed in this context by exercise-related fatigue accumulation during the 606 kicks performed by each person, individual foot-kicking patterns at the maximal and submaximal intensities could be clearly distinguished. Although all participants applied training approaches that were oriented on a collective (person-independent) profile, all ended up adopting their own individual kicking patterns that appeared to be fairly resistant against perturbations like fatigue-related changes. This indicates that, in the sense of the theory of system dynamics (Schöner and Kelso, 1988), each individual participant developed individual kicking patterns via a rather less–self-organized process. Individual movement patterns could be reproduced with a certain degree of fluctuations in different situations and influences. However, because the movement patterns were described by means of kinematic variables, no information about the kinetic changes was available. Further insight into whether the movement pattern adaptations become more or less effective by taking more or less advantage of gravitational and inertial forces is required.

### Fatigue-Related Changes in Individual Movement Patterns Across Different Timescales

Within the range of individual movement patterns, fatigue-related changes in terms of disjunct changes could be distinguished using classification analysis. Despite a large inter-individual variance in physiological and psychological variables, all participants showed significant increases in these variables over the period under study. Recurrent HR values were above 90% of the theoretical HR maximum, blood lactate concentrations were at a maximum of almost 5 mmol ^∗^ l^–1^ and the RPE fell between “hard” and “very hard.” These results confirm that the participants were highly motivated and that fatigue accumulated during the exploration. The classification of the 27 blocks (three blocks within each of the nine sets), which represent the entire fatigue-accumulating process continuously, showed a prediction accuracy of 42.9% each for the kicks with the left and the right legs. The prediction accuracies reached well above the random baseline accuracy of 3.7% and thereby suggested that the movement patterns of the respective 27 blocks could be distinguished from each other. When comparing the predicted and true blocks, shown in Figure 3A, it is noticeable that, for most misclassifications, a directly adjacent block was predicted. The increased misclassification of movement patterns of adjacent blocks of the true block further indicates that the movement patterns of kicks of adjacent blocks exhibit more similar patterns than those of kicks of more distant blocks. This observation is illustrated in Figure 4A, where the quantification of misclassifications is represented by the distance to the true class. Here, a distance of one means that, for example, the movement patterns of a kick within the sixth block was predicted to also be found in the fifth or seventh block, while, if the distance is three, the third or ninth block was similar accordingly. The misclassifications of the movement patterns of the kicks of a block decrease the further away the block is from the true block. Surprisingly, however, this general trend is interrupted by a brief increase in misclassifications for those blocks that are three or multiples of three blocks away from the true block. Interestingly, a distance of three blocks corresponds to the same block only in the adjacent set (e.g., the first block in set 3 to the first block in set 2 and the first block in set 4) and a distance of six accordingly corresponds to the same block in the sets after next (e.g., the first block in set 3 to the first block in set 1 and the first block in set 5).

The results of the classification, therefore, suggest that the movement patterns of the blocks contain common patterns within the sets, although the movement patterns seem to evolve over the entire course of the sets. Nevertheless, differentiating the movement patterns of the blocks was possible, despite that the time interval between two blocks was very short (Figure 2). However, it remains unclear how the movement patterns, which occur on timescales of a few tens of seconds, originate. An explanation of the recovery of the movement system seems highly unlikely since the 90-s break provides some recovery but was not sufficient for a full recovery. An explanation of the rhythm of movement would be more likely. These breaks within the rhythm could be caused by executing the kicks with maximal intensity at the end of each set, which could cause a kind of “reset” of the submaximal kicks. It is also possible, however, that the 90-s break alone would be enough to achieve a similar effect. A short break in rhythm could allow the subsystems of the body responsible for changing the movement patterns on timescales of tens of seconds to recover or rebuild (MacPherson et al., 2009). In this context, the influence of interruptions in the rhythmic structure on the adaptations of movement patterns, as well as their effects on training processes and outcomes, requires further research.

The trend on a timescale of tens of minutes is confirmed by the results of the classification of the movement patterns of the nine sets. The prediction accuracy values of 66.2% for left kicks and 65.1% for right kicks indicate that the movement patterns within a person can be fairly distinguished between sets (random baseline accuracy: 11.1%). Furthermore, the classifications in relation to the distance to the true set (Figure 4B) show that the misclassifications clearly decrease with the increasing distance of the set from the true set. With an error tolerance of one set, the prediction rates would already be around 90%. The similarities on a timescale of several tens of seconds, that is, the similarity of movement patterns of the blocks within different sets, are also confirmed by the classification of the three blocks within all sets. Prediction accuracy values of 55.6% for the left kicks and 66.0% for the right kicks also point to a common pattern inherent in movement patterns, although the significantly higher random baseline accuracy (33.3%) should be taken into account here.

The results of the classification analysis suggest that, during a single training session, the execution of movements seems to adapt immediately to changing psycho-physiological conditions (increasing fatigue-related changes). More specifically, even with experts, it seems that repeated executions of a ballistic sports movement (associated with exercise-related fatigue accumulation) lead to disjunct changes within individual movement patterns on different timescales (Newell et al., 2006; Schöllhorn et al., 2009; Horst et al., 2017a). The disjunct changes of individual movement patterns during exercise-related fatigue accumulation in a training session indicate continuous dynamic adaptation processes of the movement system (Schöner and Kelso, 1988). In consequence, a continuous change of the intrinsic dynamics can be assumed in parallel. While prior studies have shown emotion-specific (Janssen et al., 2008) or time-specific (Horst et al., 2016, 2017a) movement patterns, these results indicate that the movement patterns of individuals are highly dependent on the situation. Due to the situative adaptation of the movement patterns of a single individual, the findings support the perspective that it is difficult—if even possible at all—to determine a single (time-independent) person-specific optimal movement pattern (Hatze, 1986; Glazier and Mehdizadeh, 2019a,b).

### Fatigue-Related Changes in Movement Patterns, Not Variance

The present results showed that individual participants, despite practicing at an expert level of performance, were unable to repeat the kinematics of a karate front kick movement identically. As shown in Figure 5, there were slight changes in the time course of the CVs of the joint angle waveforms of the blocks and sets, although no specific trend could be identified. The variance in the movement kinematics of individuals, therefore, constantly fluctuates within a certain range. A statistically significant difference was noticed only in a period of tens of minutes (sets), between sets 4 and 5, where a decrease in the variance could be observed. However, due to the individual variance in movements across the sets, it is difficult to speak of a global trend but rather of local fluctuation. An increase in the short-term movement variance due to fatigue accumulation could not be shown within the sets. Together with the results of the classification, this leads to the conclusion that fatigue accumulation does not change the short-term movement-patterns variance but, more importantly, does change the overall kinematic movement patterns of a participant. This finding contradicts the results of previous studies (Côté, 2014; Mudie et al., 2017).

With additional evidence sourced from other research about the individuality of gait (Horst et al., 2017b, 2019), the optimal term should be considered to be individual. However, the results do show clear intra-individual shifts in movement patterns during training, which also calls into question the existence of a person-specific optimal movement pattern (Glazier and Mehdizadeh, 2019a,b). The results confirm the findings of the study by Aragonés et al. (2018), which already indicated altered movement patterns exist within individual kinematic variables. The results also are aligned with Quinzi et al. (2016) finding that there are movement pattern variations that occur even during the first 20 executions. Our results support the idea that, in this type of sportive action, the kinematic changes that occur with the accumulation of fatigue are temporary changes that span different timescales (Aragonés et al., 2013; Balagué et al., 2014). Aligned with changes at the task level, products of an evolving set of dynamic subsystems occur at multiple levels of analysis, each of which has its own timescale (Newell et al., 2009). Furthermore, the results of this study support the many findings of previous assessments of the stability and adaptability of movement concerning biomechanical forces (Newell et al., 1989; Schneider et al., 1989; Van Emmerik and Van Wegen, 2000; Bartlett, 2007). That is, the kinematic movement patterns of expert athletes are characterized by their ability to constantly adapt to new situations or intrinsic and extrinsic influences. Moreover, despite the situatedness, the movement patterns of an athlete are so individual that they clearly differ from those of other athletes.

### Practical Implications

The findings of this study support far-reaching practical implications for sports science and training. The results delivered a fairly good separation of the movement patterns of blocks, which became even clearer with increasing time. This can be associated with different fluctuations at two different timescales. One timescale is related to the duration of blocks, while the other is linked to the duration of the whole series. An additional timescale has been associated with shifts of individual movement patterns at the timescale of years (Bauer and Schöllhorn, 1997; Horst et al., 2017b) and further timescales up to ontogenetic maturation and aging can be assumed. Looking at the timescales as outcome of naturally occurring fluctuations of different amplitudes and structure resulting from repeated executions of the same movement task, it can be derived as a practical consequence that repetitive (Gaulhofer and Streicher, 1924; Anochin, 1935; Miller et al., 1960; Gentile, 1972) and variable (Schmidt, 1975; Shea and Morgan, 1979; Newell, 1986; Davids et al., 2008) sports training approaches, which are understood in terms of person and time-independent fluctuations, need to be reconsidered carefully. This reconsideration concerns the definition of target profiles as orientation for movement learning and sports training, the diagnoses of the athlete’s momentary performance, and their approximation to each other during the training process.

While the search for optimal movement solutions as target profiles in majority has been associated with a static, albeit individual, optimum movement pattern that seems to be impossible to define and to achieve (Hatze, 1986; Loeb, 2012; Glazier and Mehdizadeh, 2019a,b), one could imagine a dynamic optimum that has to be adjusted at every moment by measuring all available variables again and again. The idea of a dynamic optimum, however, also leads to the difficulty that even if the initial conditions are “completely” identified, the subsequent movement will modify the individual’s variables due to the biological memory (Walker, 1972) of the movement system and, consequently, the outcome can no more be validated due to the irreversibility of biological systems. Moreover, a dynamic, time-dependent optimum would raise ample difficulties related to the target profile and the training athlete. In the first case, the difficulty is to decide which of the fluctuating patterns should serve as an orientation for training, and in the second case, the low probability of coherent fluctuations between the dynamic target profile and the athlete’s fluctuating movement patterns will hardly allow to find a reliable intervention strategy. With regard to these issues, approaches that foster self-organized learning such as, for example, the differential learning approach (Schöllhorn, 2000), which take into account person-specific and timescale-dependent fluctuations and introduce variations without direct or indirect target profiles seem advantageous. The differential learning approach suggests increasing fluctuations in order to destabilize the movement system, thereby enabling self-organizing optimization processes (that do not require information about target profiles) (Schöllhorn et al., 2001). In this context, Schöllhorn et al. (2001) were able to find first indications of a greater extent of individualization in a group of juvenile sprinters after 6 months of training with increased fluctuations [according to the differential learning approach (Schöllhorn, 2000)] and without information about the ideal execution of movements, compared to a control group that trained according to a collective profile with error correction [according to the repetitive training approach (Jonath et al., 1995)].

The identified timescales also provide evidence of a continuously changing movement system that can be associated with the arrow of time. Apparently, athletes not only become accustomed to certain movements and, therefore, experience incremental learning over time, but also they can acclimate to a certain amount of fluctuations that blunt the sensitivity to the applied movement learning and training approach. In sports practice, it is speculated that, after a certain time of variable training, a period of repetitive training makes the movement system more sensitive to variable training again. In this context, special attention should be paid to the difference between finding an adequate description variable and assessing its impact on the training process by means of versatile types of interventions.

The amount of naturally occurring fluctuations during repetitive movements have already been considered for predicting the success of learning progress (Wu et al., 2014; Dhawale et al., 2017, 2019; Pacheco et al., 2020). Those fluctuations, however, are associated with a kind of passive dependence on the fluctuations momentarily produced by the athlete. Alternatively, the active application of subthreshold fluctuations at the foot soles led to improved posture performance (Collins et al., 1995; Priplata et al., 2002). Another type of active intervention that is based on the amplification of observed fluctuations according to the dynamic principles of systems provides promising results useful toward attaining a shortened training process (Schöllhorn, 1999) and boosting the potential for good sustainability after the intervention (Frank et al., 2008; Schöllhorn et al., 2009). How to take advantage of the passively occurring fluctuations to optimize the active fluctuations in the form of interventions demands more investigation.

In addition, the observed fluctuations in different timescales should lead us to reconsider the often-interpreted disadvantage of fatigue for movement learning. For example, the movement fluctuations that occur during the fatigue process in training could be used beneficially for movement learning. From a system dynamics view, fatigue could be considered as a type of fluctuation occurring across different timescales. With a growing focus on the individuality of movements and the sensitivity of training approaches, the situation of athletes engaged in profile-oriented sports training particularly is repeatedly disregarded and should experience an increased focus (Schöllhorn and Horst, 2019). Thereby, the detrimental effect of endurance-like training on the biomechanics of fast-contracting muscles may not be forgotten (Wilson et al., 2012).

Concerning the latent assumptions of acquisition of movement patterns, the individual, as well as the constantly fluctuating and shifting movement patterns, strongly tests the validity of the philosophy underlying repetitive sports training that is guided by collective profiles (Gaulhofer and Streicher, 1924; Anochin, 1935; Miller et al., 1960; Gentile, 1972). Theoretically, the orientation on target profiles *per se* could be detrimental for learning, regardless of whether the profiles are individual or collective. More differentiated intervention studies are required to decide whether the underlying training philosophy of collective (person-independent) profiles is deficient or whether the mostly accompanied repetitive learning approach is questionable. In the same context, whether training targeted toward an individually optimized profile will lead to individual movement patterns or at least will achieve those with less effort deserves attention. Having profiles in mind supports the disposition for comparison, which drags mental resources and increases the probability of frustration (Fillauer et al., 2020). As a consequence, this could suggest the need to move toward an alternative approach that is not oriented on set targets as in the closed-loop approach to learning (Adams, 1987) but instead fosters constant changes that support approaches originating from Far Eastern philosophy (Purser et al., 2016; Gallicchio and Ring, 2019) and which help one to be in the moment in order to achieve a brain state that is optimal for performing and learning (Henz and Schöllhorn, 2018). Being in the moment can be associated with the term situatedness as it is understood in pragmatism under contextuality (Dewey et al., 1982) or in phenomenology under situativity (Heidegger, 1927; Merleau-Ponty, 1945).

It can be expected that, with increasing the precision of measurement, tools for analysis of the unknown complexity (from a physics point of view) will be continuously decomposed and deployed in other regions or levels of interest. In combination with the knowledge about the sensitive dependence of a complex system’s (from a physics point of view) development on its initial boundary conditions, we should be careful not to reawaken the Laplace demon and accumulate endless constraints. Considering the vast amount of possible variables of influence, reaching from historical and sociocultural up to physiological and genetic conditions as well as considering their interactions according to gravitational forces and epigenetics only provides a coarse impression in the undertaking to find key variables or key exercises that are independent of individuals and timescales as held out the prospect by movement learning approaches such as the constraints-led approach (Handford et al., 1997; Davids et al., 2004, 2008; Renshaw et al., 2019).

In consequence, on a rather biological level, the goal of training practices is to focus more on adaptation processes and consider the stabilization of movement patterns rather as a byproduct. Instead of discussing stability and flexibility colloquially as complementary opposites that the athlete and coach must balance (Hamill et al., 1999; Schöllhorn, 2000; Van Emmerik and Van Wegen, 2000; Schmidt and Lee, 2005; Bartlett et al., 2007; Glazier and Davids, 2009; Schöllhorn et al., 2009; Preatoni et al., 2013), a differentiated focus related to the description of a multitude of adaptation processes dependent on different timescales (Kelso et al., 1987; Newell et al., 2001) seems more adequate to the applied problems. An increased emphasis on timescale-dependent adaptation processes in training could also have a positive effect on competition practice. The assumption is likely that highly variable and varied training also improves adaptability in competition, whether it is quick adaptation to opponents, environmental conditions, or the compensation of fatigue through changes in movement patterns.

### Limitations and Future Work

This study examined changes in the kinematic movement patterns of expert athletes who performed the karate front kick multiple times during a single training session under an accumulation of fatigue. Whether these results can be transferred to other whole-body movements requires further research. Due to the biomechanical basis of this study, the multiple underlying physiological or psychological fatigue processes can only be speculated about (Pyne and Martin, 2011; Halson, 2014). Whether the identified changes provoked by the accumulated fatigue are caused by muscular, neuronal, metabolism, or psychological mechanisms or—most probably—a mixture of everything on different timescales demands further research. However, alterations in metabolic parameters like the increased lactate and HR levels by the end of the training session indicate at least fatiguing processes occurred in all athletes. Even if the lactate values of a maximum median of almost 5 mmol ^∗^ l^–1^ are not excessively high, the HR and the RPE clearly showed that the training was carried out at a high level of intensity and that fatigue accumulated over time. An interesting problem to pursue thereby would be the possibility of an assignment or decomposition of the situative fluctuations of the movement patterns to different timescales to specific fatigue mechanisms.

An additional limitation is related to the kicks having been performed in the air. In training and competitive karate, two major forms are executed. One is the fight against a virtual opponent, called kata, and mainly consists of a series of prescribed defense and offense movements, whereas, in the second form, called kumite, a fight against a real opponent where selected hits are scored is performed. Because of the original intention to ensure high external validity with the kata form, no transfer to kicks toward an object that would be related to *kumite* could be made. Consistent with the research by Błaszczyszyn et al. (2019), it can be speculated that kicking against resistance has a considerable effect on muscle contractions, especially by the end of the movement. Further research is necessary to discern whether this also influences inter- and intra-personal differences in the fatigue-related temporal change of the karate front kick.

The present study also examined the joint angle waveforms, mainly of the lower extremities. Additional attention should be paid to discern to what extent the upper extremities also have an effect on the movement pattern recognition by experts and thus possibly improve the intra-individual recognition. However, based on the selected variables from lower extremities, similar to in gait studies (Schöllhorn et al., 2002; Janssen et al., 2008; Horst et al., 2016), clear individual movement patterns already could be found. Furthermore, it was also determined that these individual patterns change from tiring out during training. To what extent these individual movement patterns change over longer periods of tiring out during training, and the nature of possible drifts outside the individual solution spaces requires further research.

## Conclusion

Based on the classification of kinematic joint angle waveforms of karate front kicks during a training session (accompanied by increased fatiguing), unique movement patterns can be identified for individual athletes. In this research, unique movement patterns of the study individuals could be identified persistently at different execution intensities (maximal and submaximal) and with increasing fatigue. Fatigue-induced changes in individual movement patterns of the athletes could be observed in the sense of disjunctive adjustments in kinematic patterns rather than an increase in variance. These fatigue-related changes occur on different timescales (i.e., blocks in tens of seconds vs. sets in tens of minutes). The findings raise the question of to what extent the targeting of sports training on profiles, no matter whether these are derived collectively or individually, is rather a theoretical consideration (search) than a practically achievable solution. The orientation of sports training toward adaptation processes and variable situations instead of achieving and automating profiles could be a promising alternative in this context.

## Data Availability Statement

The data supporting the conclusions of this article will be made available by the authors, without undue reservation, to any qualified researcher.

## Ethics Statement

The studies involving human participants were reviewed and approved by Ethics Committee of the Medical Association of Rhineland-Pfalz (Ethik-Kommission bei EK LÄK RLP) Deutschhausplatz 3 D-55116 Mainz Rheinland-Pfalz Germany. The patients/participants provided their written informed consent to participate in this study.

## Author Contributions

DA and WS designed the experiments. AE and DA recorded and preprocessed the data. JB, FH, and WS conceived the presented idea. JB and FH performed the data analysis and designed the figures. JB, FH, and WS wrote the manuscript. JB, FH, DA, AE, and WS reviewed and approved the final manuscript. All authors contributed to the article and approved the submitted version.

## Conflict of Interest

The authors declare that the research was conducted in the absence of any commercial or financial relationships that could be construed as a potential conflict of interest.
