# Supplementary material for: Fatigue-Related and Timescale-Dependent Changes in Individual Movement Patterns Identified Using Support Vector Machine
Source: Front Psychol. 2020 Sep 30;11:551548. doi: 10.3389/fpsyg.2020.551548 (PMC7554555; doi:10.3389/fpsyg.2020.551548)
Supplement: Supplementary file 1 [file Table_1.DOCX]

Supplementary Material

# Supplementary Tables

Supplementary Table S1. Statistical analysis of HR, lactate blood concentration, and RPE.

| **Parameter** | **Friedman ANOVA** | **Post hoc analysis** | | | |
| --- | --- | --- | --- | --- | --- |
|  |  | **Comparison** | **Z** | **p** | **r** |
| **HR** | *χ^2^*(11) = 133.520, *p* < .001 | Baseline vs. Pre-Set | -3.517 | .00044* | .433 |
|  |  | Baseline vs. Set 1 | -3.517 | .00044* | .433 |
|  |  | Baseline vs. Set 2 | -3.516 | .00044* | .433 |
|  |  | Baseline vs. Set 3 | -3.518 | .00043* | .433 |
|  |  | Baseline vs. Set 4 | -3.518 | .00043* | .433 |
|  |  | Baseline vs. Set 5 | -3.516 | .00044* | .433 |
|  |  | Baseline vs. Set 6 | -3.517 | .00044* | .433 |
|  |  | Baseline vs. Set 7 | -3.516 | .00044* | .433 |
|  |  | Baseline vs. Set 8 | -3.517 | .00044* | .433 |
|  |  | Baseline vs. Set 9 | -3.517 | .00044* | .433 |
|  |  | Baseline vs. Post-Set | -3.517 | .00044* | .433 |
|  |  | Pre-Set vs. Set 1 | -3.518 | .00043* | .433 |
|  |  | Pre-Set vs. Set 2 | -3.518 | .00043* | .433 |
|  |  | Pre-Set vs. Set 3 | -3.517 | .00044* | .433 |
|  |  | Pre-Set vs. Set 4 | -3.517 | .00044* | .433 |
|  |  | Pre-Set vs. Set 5 | -3.520 | .00043* | .433 |
|  |  | Pre-Set vs. Set 6 | -3.517 | .00044* | .433 |
|  |  | Pre-Set vs. Set 7 | -3.519 | .00043* | .433 |
|  |  | Pre-Set vs. Set 8 | -3.517 | .00044* | .433 |
|  |  | Pre-Set vs. Set 9 | -3.517 | .00044* | .433 |
|  |  | Pre-Set vs. Set Post-Set | -1.889 | .05890 | .233 |
|  |  | Set 1 vs. Set 2 | -3.521 | .00043* | .433 |
|  |  | Set 1 vs. Set 3 | -3.521 | .00043* | .433 |
|  |  | Set 1 vs. Set 4 | -3.469 | .00052* | .427 |
|  |  | Set 1 vs. Set 5 | -3.519 | .00043* | .433 |
|  |  | Set 1 vs. Set 6 | -3.466 | .00053* | .427 |
|  |  | Set 1 vs. Set 7 | -3.215 | .00131* | .396 |
|  |  | Set 1 vs. Set 8 | -3.364 | .00077* | .414 |
|  |  | Set 1 vs. Set 9 | -3.466 | .00053* | .427 |
|  |  | Set 1 vs. Post-Set | -3.465 | .00053* | .427 |
|  |  | Set 2 vs. Set 3 | -3.136 | .00171 | .386 |
|  |  | Set 2 vs. Set 4 | -2.054 | .04002 | .253 |
|  |  | Set 2 vs. Set 5 | -2.796 | .00517 | .344 |
|  |  | Set 2 vs. Set 6 | -2.385 | .01710 | .294 |
|  |  | Set 2 vs. Set 7 | -1.967 | .04919 | .242 |
|  |  | Set 2 vs. Set 8 | -2.485 | .01297 | .306 |
|  |  | Set 2 vs. Set 9 | -2.873 | .00406 | .354 |
|  |  | Set 2 vs. Post-Set | -3.517 | .00044* | .433 |
|  |  | Set 3 vs. Set 4 | -0.917 | .35899 | .113 |
|  |  | Set 3 vs. Set 5 | -1.794 | .07285 | .221 |
|  |  | Set 3 vs. Set 6 | -1.223 | .22139 | .151 |
|  |  | Set 3 vs. Set 7 | -1.621 | .10502 | .200 |
|  |  | Set 3 vs. Set 8 | -1.893 | .05831 | .233 |
|  |  | Set 3 vs. Set 9 | -2.558 | .01053 | .315 |
|  |  | Set 3 vs. Post-Set | -3.517 | .00044* | .433 |
|  |  | Set 4 vs. Set 5 | -2.146 | .03187 | .264 |
|  |  | Set 4 vs. Set 6 | -1.569 | .11668 | .193 |
|  |  | Set 4 vs. Set 7 | -1.718 | .08580 | .211 |
|  |  | Set 4 vs. Set 8 | -2.191 | .02848 | .270 |
|  |  | Set 4 vs. Set 9 | -2.934 | .00334 | .361 |
|  |  | Set 4 vs. Post-Set | -3.517 | .00044* | .433 |
|  |  | Set 5 vs. Set 6 | -0.856 | .39195 | .105 |
|  |  | Set 5 vs. Set 7 | -0.703 | .48219 | .087 |
|  |  | Set 5 vs. Set 8 | -0.797 | .42531 | .098 |
|  |  | Set 5 vs. Set 9 | -1.730 | .08360 | .213 |
|  |  | Set 5 vs. Post-Set | -3.518 | .00043* | .433 |
|  |  | Set 6 vs. Set 7 | -0.398 | .69044 | .049 |
|  |  | Set 6 vs. Set 8 | -1.210 | .22621 | .149 |
|  |  | Set 6 vs. Set 9 | -3.083 | .00205 | .379 |
|  |  | Set 6 vs. Post-Set | -3.517 | .00044* | .433 |
|  |  | Set 7 vs. Set 8 | -1.483 | .13806 | .183 |
|  |  | Set 7 vs. Set 9 | -2.586 | .00971 | .318 |
|  |  | Set 7 vs. Post-Set | -3.517 | .00044* | .433 |
|  |  | Set 8 vs. Set 9 | -2.116 | .03434 | .260 |
|  |  | Set 8 vs. Post-Set | -3.517 | .00044* | .433 |
|  |  | Set 9 vs. Post-Set | -3.517 | .00044* | .433 |
| **Lactate blood concentration** | *χ^2^*(11) = 77.768, *p* < .001 | Baseline vs. Pre-Set | -3.469 | .00052* | .427 |
|  |  | Baseline vs. Set 1 | -3.517 | .00044* | .433 |
|  |  | Baseline vs. Set 2 | -3.517 | .00044* | .433 |
|  |  | Baseline vs. Set 3 | -3.517 | .00044* | .433 |
|  |  | Baseline vs. Set 4 | -3.517 | .00044* | .433 |
|  |  | Baseline vs. Set 5 | -3.518 | .00043* | .433 |
|  |  | Baseline vs. Set 6 | -3.517 | .00044* | .433 |
|  |  | Baseline vs. Set 7 | -3.517 | .00044* | .433 |
|  |  | Baseline vs. Set 8 | -3.517 | .00044* | .433 |
|  |  | Baseline vs. Set 9 | -3.517 | .00044* | .433 |
|  |  | Baseline vs. Post-Set | -3.519 | .00043* | .433 |
|  |  | Pre-Set vs. Set 1 | -3.233 | .00123 | .398 |
|  |  | Pre-Set vs. Set 2 | -3.286 | .00102 | .404 |
|  |  | Pre-Set vs. Set 3 | -3.130 | .00175 | .385 |
|  |  | Pre-Set vs. Set 4 | -3.078 | .00209 | .379 |
|  |  | Pre-Set vs. Set 5 | -3.103 | .00192 | .382 |
|  |  | Pre-Set vs. Set 6 | -3.155 | .00160 | .388 |
|  |  | Pre-Set vs. Set 7 | -3.104 | .00191 | .382 |
|  |  | Pre-Set vs. Set 8 | -3.206 | .00134 | .395 |
|  |  | Pre-Set vs. Set 9 | -3.233 | .00123 | .398 |
|  |  | Pre-Set vs. Set Post-Set | -2.302 | .02132 | .283 |
|  |  | Set 1 vs. Set 2 | -1.578 | .11452 | .194 |
|  |  | Set 1 vs. Set 3 | -1.268 | .20475 | .156 |
|  |  | Set 1 vs. Set 4 | -1.217 | .22369 | .150 |
|  |  | Set 1 vs. Set 5 | -1.605 | .10859 | .198 |
|  |  | Set 1 vs. Set 6 | -1.677 | .09357 | .206 |
|  |  | Set 1 vs. Set 7 | -1.364 | .17259 | .168 |
|  |  | Set 1 vs. Set 8 | -1.811 | .07018 | .223 |
|  |  | Set 1 vs. Set 9 | -1.889 | .05886 | .233 |
|  |  | Set 1 vs. Post-Set | -0.931 | .35182 | .115 |
|  |  | Set 2 vs. Set 3 | -1.191 | .23354 | .147 |
|  |  | Set 2 vs. Set 4 | -0.777 | .43720 | .096 |
|  |  | Set 2 vs. Set 5 | -1.139 | .25473 | .140 |
|  |  | Set 2 vs. Set 6 | -1.397 | .16239 | .172 |
|  |  | Set 2 vs. Set 7 | -0.967 | .33330 | .119 |
|  |  | Set 2 vs. Set 8 | -1.165 | .24391 | .143 |
|  |  | Set 2 vs. Set 9 | -1.847 | .06475 | .227 |
|  |  | Set 2 vs. Post-Set | -1.591 | .11162 | .196 |
|  |  | Set 3 vs. Set 4 | -0.026 | .97933 | .003 |
|  |  | Set 3 vs. Set 5 | -0.505 | .61359 | .062 |
|  |  | Set 3 vs. Set 6 | -1.280 | .20055 | .158 |
|  |  | Set 3 vs. Set 7 | -0.659 | .50959 | .081 |
|  |  | Set 3 vs. Set 8 | -1.005 | .31506 | .124 |
|  |  | Set 3 vs. Set 9 | -1.705 | .08821 | .210 |
|  |  | Set 3 vs. Post-Set | -2.160 | .03077 | .266 |
|  |  | Set 4 vs. Set 5 | -0.338 | .73547 | .042 |
|  |  | Set 4 vs. Set 6 | -1.399 | .16169 | .172 |
|  |  | Set 4 vs. Set 7 | -0.171 | .86454 | .021 |
|  |  | Set 4 vs. Set 8 | -0.483 | .62919 | .059 |
|  |  | Set 4 vs. Set 9 | -1.682 | .09258 | .207 |
|  |  | Set 4 vs. Post-Set | -1.966 | .04931 | .242 |
|  |  | Set 5 vs. Set 6 | -1.570 | .11646 | .193 |
|  |  | Set 5 vs. Set 7 | -0.311 | .75593 | .038 |
|  |  | Set 5 vs. Set 8 | -0.905 | .36528 | .111 |
|  |  | Set 5 vs. Set 9 | -1.708 | .08772 | .210 |
|  |  | Set 5 vs. Post-Set | -2.174 | .02971 | .268 |
|  |  | Set 6 vs. Set 7 | -0.466 | .64105 | .057 |
|  |  | Set 6 vs. Set 8 | -0.284 | .77616 | .035 |
|  |  | Set 6 vs. Set 9 | -1.268 | .20475 | .156 |
|  |  | Set 6 vs. Post-Set | -2.457 | .01399 | .302 |
|  |  | Set 7 vs. Set 8 | -1.348 | .17765 | .166 |
|  |  | Set 7 vs. Set 9 | -0.995 | .31996 | .122 |
|  |  | Set 7 vs. Post-Set | -2.502 | .01236 | .308 |
|  |  | Set 8 vs. Set 9 | -0.256 | .79787 | .032 |
|  |  | Set 8 vs. Post-Set | -2.732 | .00629 | .336 |
|  |  | Set 9 vs. Post-Set | -2.794 | .00521 | .344 |
| **RPE** | *χ^2^*(11) = 161.988, *p* < .001 | Baseline vs. Pre-Set | -3.530 | .00042* | .435 |
|  |  | Baseline vs. Set 1 | -3.534 | .00041* | .435 |
|  |  | Baseline vs. Set 2 | -3.555 | .00038* | .438 |
|  |  | Baseline vs. Set 3 | -3.594 | .00033* | .442 |
|  |  | Baseline vs. Set 4 | -3.537 | .00040* | .435 |
|  |  | Baseline vs. Set 5 | -3.542 | .00040* | .436 |
|  |  | Baseline vs. Set 6 | -3.533 | .00041* | .435 |
|  |  | Baseline vs. Set 7 | -3.545 | .00039* | .436 |
|  |  | Baseline vs. Set 8 | -3.532 | .00041* | .435 |
|  |  | Baseline vs. Set 9 | -3.530 | .00042* | .435 |
|  |  | Baseline vs. Post-Set | -3.423 | .00062* | .421 |
|  |  | Pre-Set vs. Set 1 | -3.329 | .00087* | .410 |
|  |  | Pre-Set vs. Set 2 | -3.436 | .00059* | .423 |
|  |  | Pre-Set vs. Set 3 | -3.438 | .00059* | .423 |
|  |  | Pre-Set vs. Set 4 | -3.543 | .00040* | .436 |
|  |  | Pre-Set vs. Set 5 | -3.541 | .00040* | .436 |
|  |  | Pre-Set vs. Set 6 | -3.544 | .00039* | .436 |
|  |  | Pre-Set vs. Set 7 | -3.545 | .00039* | .436 |
|  |  | Pre-Set vs. Set 8 | -3.544 | .00039* | .436 |
|  |  | Pre-Set vs. Set 9 | -3.536 | .00041* | .435 |
|  |  | Pre-Set vs. Set Post-Set | -1.371 | .17045 | .169 |
|  |  | Set 1 vs. Set 2 | -3.541 | .00040* | .436 |
|  |  | Set 1 vs. Set 3 | -3.541 | .00040* | .436 |
|  |  | Set 1 vs. Set 4 | -3.532 | .00041* | .435 |
|  |  | Set 1 vs. Set 5 | -3.449 | .00056* | .425 |
|  |  | Set 1 vs. Set 6 | -3.440 | .00058* | .423 |
|  |  | Set 1 vs. Set 7 | -3.443 | .00058* | .424 |
|  |  | Set 1 vs. Set 8 | -3.213 | .00132* | .395 |
|  |  | Set 1 vs. Set 9 | -3.115 | .00184* | .383 |
|  |  | Set 1 vs. Post-Set | -1.722 | .08502 | .212 |
|  |  | Set 2 vs. Set 3 | -2.000 | .04550 | .246 |
|  |  | Set 2 vs. Set 4 | -3.207 | .00134* | .395 |
|  |  | Set 2 vs. Set 5 | -3.272 | .00107* | .403 |
|  |  | Set 2 vs. Set 6 | -3.453 | .00055* | .425 |
|  |  | Set 2 vs. Set 7 | -3.594 | .00033* | .442 |
|  |  | Set 2 vs. Set 8 | -3.543 | .00040* | .436 |
|  |  | Set 2 vs. Set 9 | -3.537 | .00040* | .435 |
|  |  | Set 2 vs. Post-Set | -2.923 | .00347* | .360 |
|  |  | Set 3 vs. Set 4 | -3.217 | .00130* | .396 |
|  |  | Set 3 vs. Set 5 | -3.345 | .00082* | .412 |
|  |  | Set 3 vs. Set 6 | -3.564 | .00037* | .439 |
|  |  | Set 3 vs. Set 7 | -3.547 | .00039* | .437 |
|  |  | Set 3 vs. Set 8 | -3.538 | .00040* | .435 |
|  |  | Set 3 vs. Set 9 | -3.060 | .00222* | .377 |
|  |  | Set 3 vs. Post-Set | -3.217 | .00130* | .396 |
|  |  | Set 4 vs. Set 5 | -2.111 | .03481 | .260 |
|  |  | Set 4 vs. Set 6 | -3.169 | .00153* | .390 |
|  |  | Set 4 vs. Set 7 | -3.236 | .00121* | .398 |
|  |  | Set 4 vs. Set 8 | -3.336 | .00085* | .411 |
|  |  | Set 4 vs. Set 9 | -3.455 | .00055* | .425 |
|  |  | Set 4 vs. Post-Set | -3.193 | .00141* | .393 |
|  |  | Set 5 vs. Set 6 | -2.887 | .00389* | .355 |
|  |  | Set 5 vs. Set 7 | -3.035 | .00241* | .374 |
|  |  | Set 5 vs. Set 8 | -3.256 | .00113* | .401 |
|  |  | Set 5 vs. Set 9 | -3.443 | .00058* | .424 |
|  |  | Set 5 vs. Post-Set | -3.420 | .00063* | .421 |
|  |  | Set 6 vs. Set 7 | -1.667 | .09558 | .205 |
|  |  | Set 6 vs. Set 8 | -2.521 | .01170 | .310 |
|  |  | Set 6 vs. Set 9 | -3.250 | .00115* | .400 |
|  |  | Set 6 vs. Post-Set | -3.525 | .00042* | .434 |
|  |  | Set 7 vs. Set 8 | -2.333 | .01963 | .287 |
|  |  | Set 7 vs. Set 9 | -3.176 | .00149* | .391 |
|  |  | Set 7 vs. Post-Set | -3.525 | .00042* | .434 |
|  |  | Set 8 vs. Set 9 | -2.714 | .00666 | .334 |
|  |  | Set 8 vs. Post-Set | -3.527 | .00042* | .434 |
|  |  | Set 9 vs. Post-Set | -3.527 | .00042* | .434 |

*Note*: Presented are the results of the Friedman ANOVA to determine differences among all classes and pairwise Wilcoxon signed rank post-hoc test. HR = heart rate, RPE = rating of perceived exertion. *significant values according to Holm-Bonferroni-corrected alpha level: α_0_ = 0.00076.
